# Understanding the Effects of Prenatal Alcohol Exposure Using Three Dimensional Facial Imaging

**Published:** 2011

**Authors:** Leah Wetherill, Tatiana Foroud

One of the (at least theoretically) most easily detectable features of fetal alcohol syndrome (FAS) and fetal alcohol spectrum disorders (FASD) is a distinct pattern of facial characteristics. However, in many children prenatally exposed to alcohol, these characteristics are expressed only subtly, making it difficult to correctly identify children with these disorders. To date, several studies have used conventional two-dimensional images to develop computerized programs assisting in the identification of individuals with FAS or FASD. However, many of the subtle features of prenatal alcohol exposure cannot be visualized using two-dimensional images. Therefore, researchers at the Collaborative Initiative on Fetal Alcohol Spectrum Disorders (CIFASD; http://www.cifasd.org) have been using a special camera system that can generate three-dimensional images, which allows them to explore the advantages of using such images to identify subtle facial differences between individuals who were exposed to alcohol prenatally and individuals who were not. This approach may help investigators and clinicians to better understand the complications that may arise from prenatal alcohol exposure. For example, CIFASD researchers can use facial measurements or shapes obtained from the three-dimensional images to predict the presence of FAS, examine associations between facial shapes and cognitive deficiencies, or better understand how the facial growth of a person with FAS compares with facial growth in someone not prenatally exposed to alcohol. Through an international consortium, CIFASD has been addressing these questions in various age groups as well as different ethnic groups.

## The Three-Dimensional Camera System and Image Analysis

**Figure 1:**
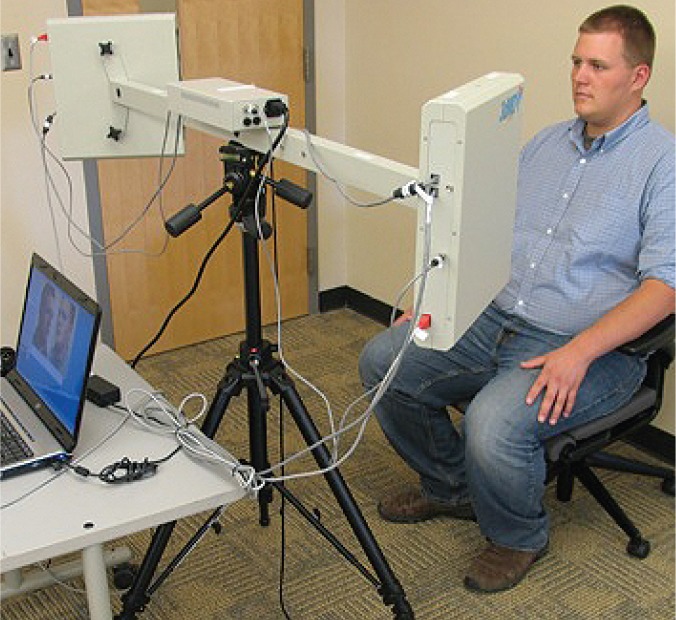
The camera system used to obtain three-dimensional images.

**Figure 2:**
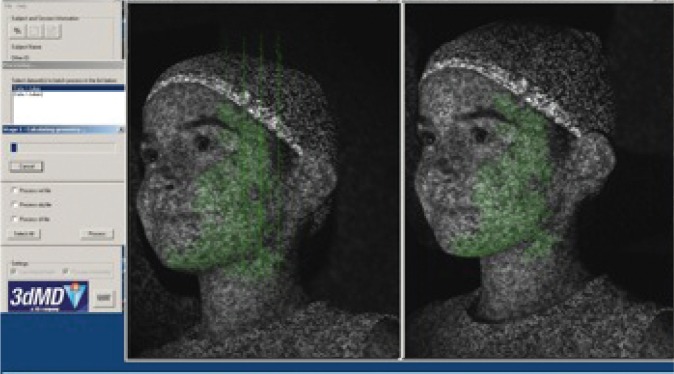
Software stitching. Example of the 3dMD proprietary software, stitching together the six images obtained from the cameras.

**Figure 3:**
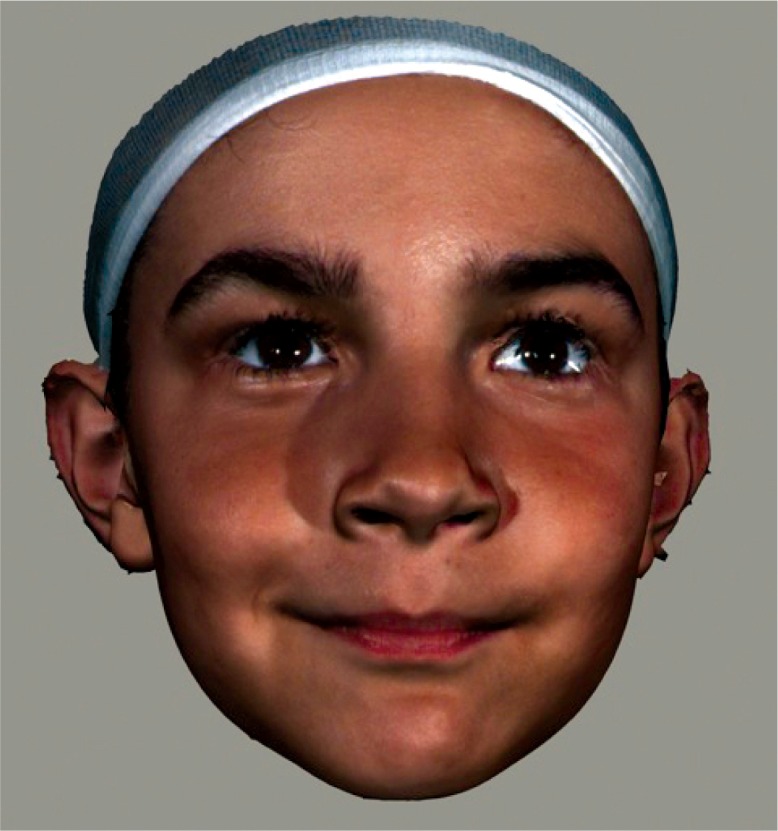
Three-dimensional image. Example of the final three-dimensional image obtained from the camera.

The camera system used to obtain the three-dimensional images consists of two pods attached at each end of a long arm that is mounted to a standard camera tripod (see figure 1). Each pod contains three cameras and two flashes so that a total of six photographs are generated. These six photographs, which eventually comprise the three-dimensional image, are obtained in 1.5 milliseconds, similar to normal flash photography. The attached laptop computer system uses special software to automatically stitch together the six photographs (see figure 2), generating the final three-dimensional image in less than 2 minutes (see figure 3). Although the camera system looks big and bulky, it can easily be taken apart and packed into two cases. As a result, CIFASD researchers have the ability to transport the camera anywhere in the world, take three-dimensional images of dozens of children within a day, and electronically transfer these images to a secure computer for analysis.

**Figure 4:**
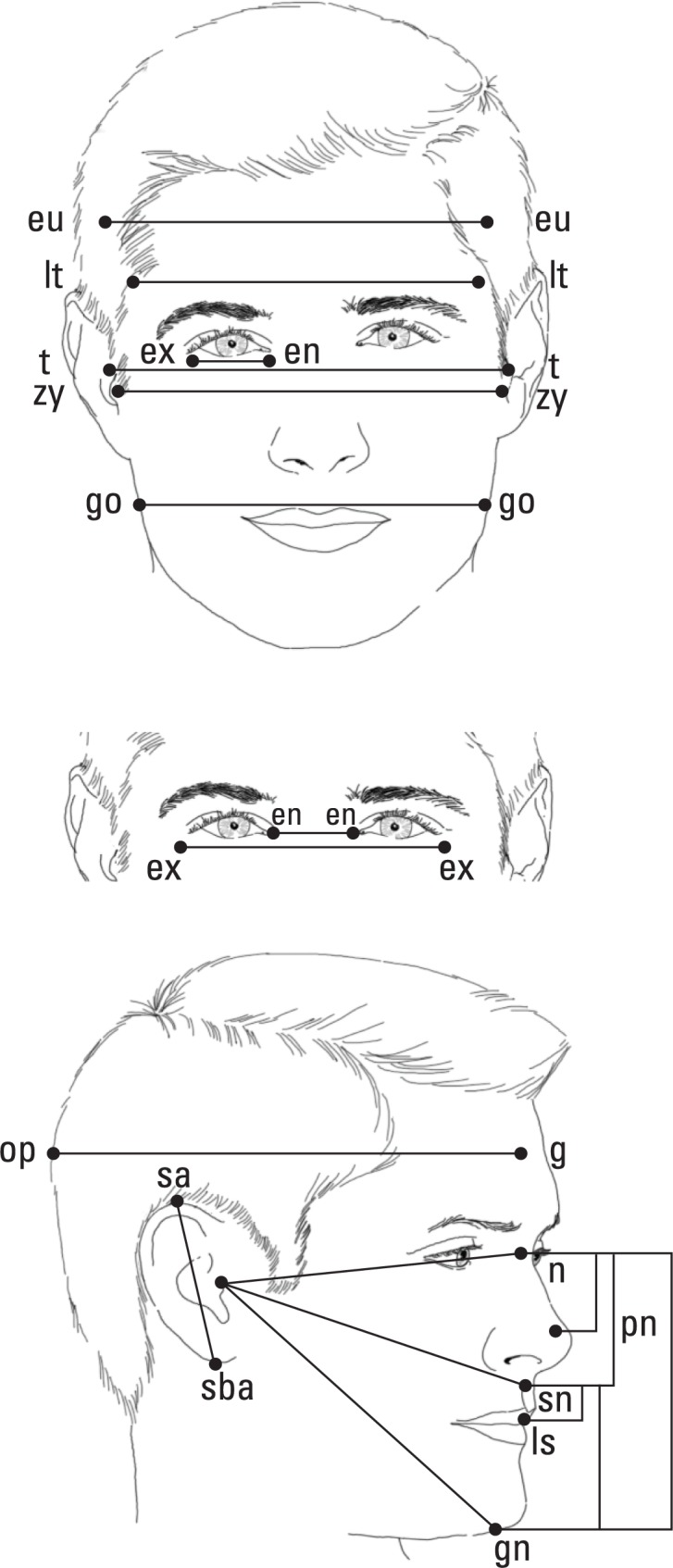
Anthropometric measurements. The 16 anthropometric measurements obtained from each of the three-dimensional images.

The three-dimensional images can be analyzed in several ways. One of the simpler analytic strategies is to measure the length, width, or height of various portions of the face, such as the length of the eye, width of the forehead, or height of the upper face (figure 4). CIFASD investigators have used these measurements to evaluate whether these parameters differ in people with and without prenatal alcohol exposure. The analyses found that by using a subset of these measurements as predictor variables in a statistical method called logistic regression, one can accurately classify children in a given sample into two groups: those with FAS and those who were not exposed to alcohol prenatally. For example, when studying children from Cape Town, South Africa, CIFASD researchers identified a set of measurements that could correctly classify 94 percent of children with FAS and 91 percent of children without prenatal alcohol exposure (Moore et al. 2007). Using the same statistical approach, but with a group of children from Helsinki, Finland, the researchers also identified a set of facial measurements that correctly classified 96 percent of children with FAS and 91 percent of those without prenatal alcohol exposure. Interestingly, the sets of facial measurements that best identified the respective groups differed between the South African and Finnish samples. However, in both groups small eye width was one of the parameters that helped to predict FAS (Moore et al. 2007). These results support previous observations that small eye widths are a key feature distinguishing individuals with FAS and without prenatal alcohol exposure.

CIFASD researchers now are seeking to understand why some unique facial features helped predict group membership in the South African and Finnish samples. One possibility is that these differences are caused by facial variation attributable to ethnicity. Alternatively, the differences may be related to age differences between the two samples because the South African children, on average, were much younger than the Finnish participants, and previous studies already noted that the facial characteristics of people with FAS change with age (Mutsvangwa et al. 2010; Streissguth et al. 1991).

**Figure 5:**
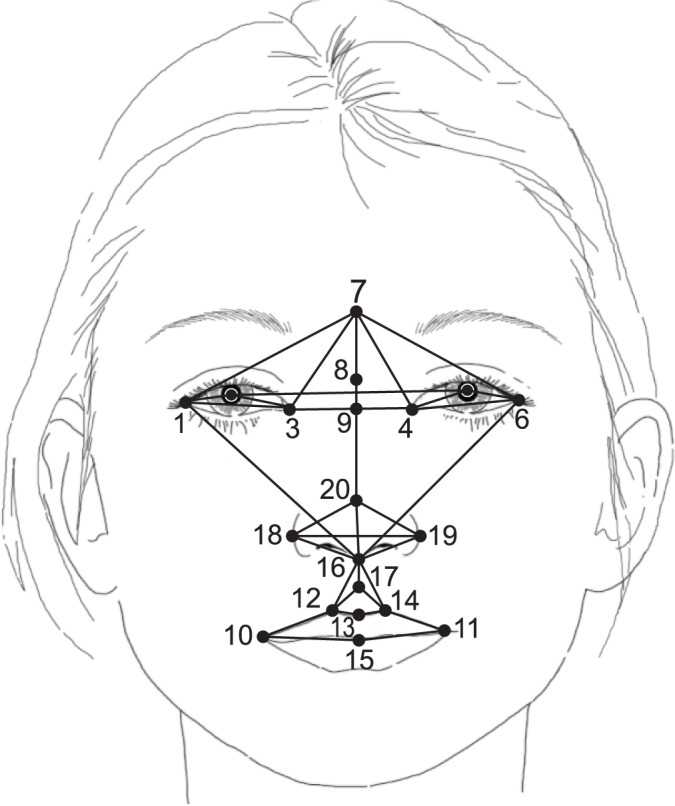
Example of shapes. Landmarks are placed in specific anthropomorphically defined places. The landmarks are then connected to create facial shapes.

Another way to analyze three-dimensional images is to place specific landmarks on the images and then connect the landmarks with lines that generate shapes (see figure 5) (Mutsvangwa and Douglas 2007), a method that originally was proposed by Clarren and colleagues (1987). Using an approach called morphometrics, one then can look at differences between the shapes found in the faces of children prenatally exposed to alcohol and those found in the faces of nonexposed children. To achieve this, the shapes obtained from the faces of all subjects are aligned statistically so that they then can be compared between the two groups (i.e., people with FAS and control individuals) (Douglas and Mutsvangwa 2010). Similarly, one can compare the shapes between younger and older individuals (Mutsvangwa et al. 2010). Using this approach, researchers can examine what information specific shapes can provide about an individual’s face—such as the fullness of the face, slower growth in certain facial areas, or asymmetries between the two sides of the face. Thus, CIFASD researchers have found that differences exist in the shape of particular facial regions between children with FAS and controls (Klingenberg et al. 2010). In addition, certain differences in facial shapes appear to be related to performance on tests that measure cognitive function (i.e., IQ) (Wetherill et al. 2009). Findings such as these are important because they begin to allow researchers to better understand how prenatal alcohol exposure affects development, both in the face and in the brain.

## Potential Applications of the Three-Dimensional Imaging System

The three-dimensional facial imaging system has several potential applications that may aid researchers and clinicians in identifying individuals with FAS and delineating the consequences of prenatal alcohol exposure. For example, the technology may allow researchers to track how the facial features associated with prenatal alcohol exposure change as an individual grows up. Studies currently are underway in a group of children seen in South Africa in 2005, when most of them were 5 years old, and then again in 2009, when they were 9 years of age. Analyzing data from both time points, CIFASD researchers found that during this time period, children with FAS exhibited faster growth than non–alcohol-exposed children in particular parts of the face. As a result, the facial features most characteristic of FAS (i.e., small eyes and small face) became larger, making the facial features less distinct (Mutsvangwa et al. 2010; Wetherill et al. 2010). This observation confirms previous reports by doctors and clinicians who specialize in the effects of prenatal alcohol exposure that the facial features of children with FAS become less obvious as the children age.

Another application currently being explored by members of CIFASD is the opportunity to study changes in the faces of younger children. To this end, CIFASD is working with a team of pediatricians at Tygerberg Hospital, a part of Stellenbosch University Medical School, South Africa, to take three-dimensional images of babies at 1 month of age and again at 12 months. The investigators hope to enroll about 1,200 babies in the study, about 600 of whom will have been prenatally exposed to alcohol. Extensive data is available on the children’s prenatal alcohol exposure, which will allow the research team to study how the babies’ faces change during the first year depending on the amount and timing of the prenatal alcohol exposure.

Finally, studies involving participants of differing ages, races, and ethnicities will allow the CIFASD team to devise new ways to identify children with prenatal alcohol exposure. Because it is essential for the children’s prognosis to initiate interventions as early as possible, this approach using the three-dimensional camera and image analysis can lead to earlier detection of and intervention for those at greatest risk.
